# Free does not mean affordable: maternity patient expenditures in a public hospital in Bangladesh

**DOI:** 10.1186/1478-7547-3-1

**Published:** 2005-01-19

**Authors:** Suhaila H Khan

**Affiliations:** 1 Department of International Health and Development, Tulane School of Public Health and Tropical Medicine, 1440 Canal Street, Suite 2200, New Orleans, LA 70112, USA

## Abstract

**Objective:**

This study investigated a) the amount and types of out-of-pocket expenditures by patients for nominally free services in a large public hospital in Bangladesh, b) the factors influencing these expenses, and c) the impact of these expenses on household income.

**Methods:**

Eighty-one maternity patients were interviewed during their hospitalization in the Dhaka Medical College Hospital. Patients were selected by quota sample to match the distribution of maternity patient categories in the hospital. Patients were interviewed with a semi-structured, in-depth questionnaire.

**Results:**

All interviewees incurred substantial out-of-pocket expenditures for travel, hospital admission fees, medicine, tests, food, and tips. Only two of the expenditures, travel expenses and admission fees, were not supposed to be provided free of charge by the hospital. The median total per-patient expenditure was $65 (range $2–$350), equivalent to 7% (range 0.04%–225%) of annual household income. Half of all patients reported that their families had to borrow to pay for care at interest rates of 5%–30% per month. A third of these families reported selling jewelry, land or household items to moneylenders. The rural patients reported more difficulty in paying for care than the urban patients. Factors increasing the expenditures were duration of hospitalization, rural residence, and necessary (e.g. C-section, hysterectomy) and unnecessary (e.g. episiotomy) medical procedures.

**Conclusion:**

Free maternity services in Bangladesh impose large out-of-pocket expenditures on patients. Authorities could reduce the burden by reducing the duration of hospital stays, limiting use of medical procedures, eliminating tips, and moving routine services closer to potential users. Fee for service could reduce unofficial expenditures if the fee were lower than and replaced typical unofficial expenditures, otherwise adding service fees without reform of current hospital practices would lead to even more burdensome expenditures and inequities.

## Background

In developing countries governments often subsidize services at public health care facilities and provide them free of charge to users. However, evidence suggests that users still incur large expenditures using the 'free' services for such things that are supposedly provided without charge. Studies have found that patients incurred substantial out-of-pocket expenditures for medicine, food and travel from the use of 'free' public health facilities [1-3]. A study in Vietnam found that out-of-pocket payments can cause serious equity problems such as the poor becoming poorer without greatly affecting the non-poor [4]. Household difficulty in payment of health care expenses can result in the 'distress sale' of property, delay or abandonment of treatment, and sacrifice expenditures on food and education [3,5]. Other studies have found that introducing or increasing user fees negatively affect the utilization of public health facilities [6-9].

Three previous studies have explored issues related to patient expenditures in Bangladesh [3,10,11]. Nahar et al. enumerated the patient expenditures and affordability of free maternity services for normal delivery and caesarean section. Killingsworth et al. explored the linkage between official and unofficial fees in public health facilities, and concluded that these fees had income and equity effects. Stanton et al. reviewed literature on user fees and pointed out the need to further investigate the factors and practices causing patient expenses before institutional implementation of user fees.

Thus, this study examined the type, amount and household financial results of out-of-pocket expenditures by patients for nominally free services in a large government hospital in Dhaka. The study also identified the factors and medical practices producing and influencing the out-of-pocket expenditures. Plans to begin fees for service in Bangladesh make it important to document the amount of money actually being paid by the patients under the present system. If current expenditures are large, fee for service may have serious negative impacts on utilization and on the economic well-being of Bangladeshi households. If current expenditures are modest, it is possible that such fees will have a lesser impact.

## Methods

### Study site

The study was conducted in the Department of Obstetrics and Gynaecology (ObGyn) of the Dhaka Medical College Hospital (DMCH). DMCH is the largest teaching hospital in Bangladesh with 850 beds located in the capital city. DMCH is government funded and provides a wide range of out- and in-patient services. Public hospitals have two payment categories for in-patients: non-paying and paying. Patients first go to an out-patient unit for diagnosis where they are categorized as out- or in-patient. Those categorized as in-patient are then classified as paying or non-paying by observing the clothes and general appearance of the woman and any accompanying relatives. Non-paying patients pay only the hospital admission fee. Paying in-patients are charged the fees for hospital admission, bed, and surgery. The various fees are: hospital admission fee: $0.23, bed fee: $1.34–3.50 per day, surgery fee: $12.50–125. Taka was converted into US dollars using the 1994 exchange rate of US$1.00 = Taka 40.00. Neither patient category is supposed to pay for medicine, tests, food, nursing and other support services during hospitalization; these commodities and services are theoretically provided free by the hospital.

### Study population, sampling and sample size

The study interviewed 81 non-paying in-patients hospitalized for reproductive health conditions (about two thirds were for maternity conditions). Patients were selected by quota sample matching the distribution of the patient categories in the hospital i.e. the selected medical conditions accounted for the greatest number of ObGyn admissions reported for the hospital, and also reflect the causes associated with high maternal mortality and morbidity in Bangladesh [12]. These included normal vaginal delivery (NVD), caesarean section (C-section), abortion, and hysterectomy. NVDs included cases with episiotomy, without episiotomy, and with eclampsia. C-sections included elective and eclamptic cases. Abortion included non-septic and septic abortions. Hysterectomies included abdominal and vaginal hysterectomies for treating fibroid, prolapsed uterus, and pelvic inflammatory disease. Table 1 illustrates the distribution of the selected cases for this study.

**Table 1:** No. of in-patients surveyed by medical condition

	**No. of patients**
**Normal Vaginal Delivery**	**19**
with episiotomy	5
without episiotomy	5
with eclampsia	9
**Caesarean section**	**20**
elective	10
eclamptic	10
**Abortion**	**20**
non-septic	10
septic	10
**Hysterectomy**	**22**
prolapsed uterus	10
fibroid	9
pelvic inflammatory disease	3
**Total**	**81**

### Variables

Information was collected on various characteristics of the study participants. Demographic characteristics included age, education, marital status, and residence. Socio-economic characteristics included occupation and annual household income. Information was also collected on underlying medical condition. Out-of-pocket expenditure related information included types and amounts of expenses incurred during hospitalization such as those for travel, medicine, food, fees, etc. Factors influencing expenditures included type of treatment received and duration of hospitalization. Sources of funds included amount borrowed and interest charged for borrowed amount.

### Data collection tools and technique

Data were collected from patients and their relatives with semi-structured open-ended questionnaires between January – June 1994. The interviewers were physicians employed in DMCH. The interviewers selected the cases by diagnosis from patient admission records. To minimize possible selection bias the first case was selected randomly from the records and then every third case was selected. The selected patients were interviewed a minimum of three times to minimize recall error. Recall error was also minimized as information was collected while patients were still hospitalized. During the first interview demographic and socio-economic information was collected with structured questions. During the second and third interviews information related to expenditures was collected with open-ended questions.

To illustrate the data collection process a description of an interview with a typical C-section patient follows. C-section patients are usually hospitalized for two weeks in DMCH. On the first day of hospitalization an interviewer collected information on patient's age, education, marital status, etc. On the eighth day of hospitalization the second interview collected information on treatment received, treatment related out-of pocket expenditures, annual household income, amount of money borrowed to pay for treatment, source of borrowed money, and interest rate charged. On the fourteenth day the third interview collected more monetary information on out-of-pocket expenditures, and on expected expenditures immediately after leaving the hospital. This survey did not cover the expenditures for the full course of the treatment. Expenditure estimates were derived for the duration of the current hospitalization only, i.e. from the day of admission until the day of discharge. Expenditures immediately before admission and after discharge from the hospital included only travel expenses to and from the hospital for the patient and her accompanying relatives.

## Results

### Socio-demographic characteristics of study participants

The median age of the study participants was 26 years (range 15–60 years). The majority (88%) of the patients were married, the rest were separated (4%), divorced (2%), and unmarried (1%). Forty-four percent of the patients lived in rural areas. The median annual household income was $750 (range $3–$6000) per respondent. The annual household income was higher for the urban (median $900; range $150–$6000) than the rural (median $615; range $3–$6000) respondents.

### Patient out-of-pocket expenditures

All 81 patients interviewed reported incurring substantial out-of-pocket expenditures during their hospitalization. These out-of-pocket expenditures were for travel, hospital admission fee, medicine, tests, food, tips, and other items. As expected there were expenditures related to travel and admission fees which the hospital is not supposed to subsidize. But there were also expenditures for medicine, tests, food, tips, and other items which were supposed to be provided free from the hospital but were not.

The median total expenditure for hospitalization was $65 (range $2.15–$350) per patient. On average, 61% of these expenditures ($49) were for services and commodities that were supposed to be provided free from the hospital but were not. The per patient median expenditure for the various expense categories were: medicine $26, tests 0, tips $1.25, food $1.25, other items $4.38, travel $22.25, and hospital admission fee $0.25. On average, medicine constituted 42%, travel 38%, tests 5%, food 4%, tips 2%, admission fees <1%, and others 8% of the total expenditures. C-section and hysterectomy cases had the highest median expenditures. Table 2 illustrates the out-of-pocket expenditures by items not supposed to be provided free by the hospital and items supposed to be given free from the hospital. A description of the expenses follows.

**Table 2:** Distribution of the out-of-pocket expenditures by medical condition (in US$) in 1994

	**Expenditures on items NOT supposed to be provided from hospital**		**Expenditures on items supposed to be provided free from hospital**					
	**Travel**	**Fee**	**Medicine**	**Food**	**Tips**	**Other**	**Tests**	**Total**

**NVD ** (n = 19)								
median	12.50	0.25	11.25	0.88	1.25	3.88	0.00	62.50
mean	29.72	0.23	18.35	2.24	2.11	5.99	3.42	62.04
range	1–127	0.10–0.33	1–70	0–23	0.75–5	0–25	0–23	6–225
**C-section ** (n = 20)								
median	36.70	0.10	51.88	2.50	2.06	11.25	0.00	118.75
mean	44.32	0.20	63.52	4.25	2.56	16.79	1.88	133.50
range	5–150	0.10–0.75	25–160	0.50–16	0.25–7	2–75	0–25	41–350
**Abortion ** (n = 20)								
median	3.08	0.18	12.06	0.84	0.63	0.00	0.00	15.56
mean	11.67	0.17	18.93	1.40	0.77	0.00	0.00	32.94
range	1–63	0–0.30	1–75	0–5	0–2	0.00	0.00	2–125
**Hysterectomy **(n = 22)								
median	23.25	0.25	36.25	2.50	1.25	5.00	0.00	75.50
mean	32.04	0.38	30.89	4.84	2.38	4.02	10.15	84.70
range	1–86	0.10–4	1–50	0–19	0–10	0–10	0–75	2–178
**Total **(N = 81)								
median	22.25	0.23	26.25	1.25	1.25	4.38	0.00	65.25
mean	29.50	0.24	33.05	3.23	1.96	6.64	4.02	78.65
range	1–150	0–4	1–160	0–23	0–10	0–75	0–75	2–350

NVD: normal vaginal delivery

#### Expenditures on items supposed to be provided free from hospital

##### Medicine

All patients were supposed to be provided required medicines free from the hospital but were not. Medicines included antibiotics, analgesics, syringe, catheter, blood, and so forth. Medicine was usually bought when patients were admitted at night. The medicine required for treatment is ordered by the on-duty physician but it takes several hours for the hospital management to process the order. Thus, no free medicine is available immediately. To start the treatment, the on-duty physician requests the patient's relatives to buy the medicine which is purchased from nearby private pharmacies.

##### Tests

All tests (e.g. pathology, radiology) are supposed to be provided by the hospital but sometimes the patients had the tests done in a private laboratory because waiting time for tests is very long in the DMCH due to the high patient load.

##### Food

Food is provided by the hospital but the interviewees found the hospital food of poor quality or totally lacking (liquid food such as soup or horlicks had to be bought for patients who had undergone surgery since these were not provided by the hospital). Relatives usually stayed with the patient in the hospital because of lack of *ayahs *(cleaning ladies) or nurses to provide necessary services. Thus, food was usually bought from a vendor or brought from home for both patient and relatives.

##### Tips

Tips (*bakshish*) are payments made to *ayahs *and guards. *Ayahs *were given tips for routine services such as pushing the patient's trolley to and from the labour/operation room, shaving the patient before delivery/surgery, giving enemas, etc. Guards at the gates were tipped each time a relative came to visit the patient during non-visitor hours. However, *ayahs *and guards are salaried hospital employees and are supposed to provide these services free of charge. The patients were reluctant when talking about the tips probably because they were still hospitalized and depended on these employees for access to certain services.

##### Other items

The other expenditures included items for the patient (e.g. hot water, bucket for hot water) and the newborn baby (e.g. blanket) that were supposed to be provided by the hospital free of charge but were not.

#### Expenditures on items not supposed to be provided by the hospital

##### Travel

Travel expenses are not supposed to be provided by the hospital. Travel expenses consisted of travel to and from the hospital by the patient and any accompanying relatives, and travel expenditures of relatives during hospitalization for purchasing medicine and food for the patient. The patients came to DMCH because they expected 'free' and 'affordable' services compared to private clinics, or they were referred from a primary/secondary level facility, or to get better treatment here. Patients from rural/peri-urban areas took longer to reach DMCH than those from urban Dhaka (range half an hour to two days).

##### Hospital admission fee

This expense is also not supposed to be covered by the hospital. The official price of admission was $0.23, but it was zero for two patients and more than the official price for half of the patients interviewed. The study could not elicit the reason the patients paid more than the official price. When probed the patients could not or would not elaborate beyond the amount paid. The patients were very reluctant when talking about paying more than the official price for the admission fee.

### Factors increasing out-of-pocket expenditures

*Duration of hospitalization *and *rural residence *of the patients increased the out-of-pocket expenditures. Rural residence increased the travel expenses and thus the total expenditures. Longer duration of hospitalization increased virtually all expenditures. The median duration of hospitalization was 8 days (range 1–34 days) per patient. Duration of hospitalization was the longest for hysterectomies followed by C-sections. Duration of hospitalization was related to severity of medical condition (e.g. eclampsia), necessary medical procedures (e.g. hysterectomy), and unnecessary medical procedures (e.g. episiotomy). One day of extra hospitalization increased expenditures by $2.30 per patient.

Choice of *medical procedures *increased the patient expenditures. *Episiotomy *increased expenditures as patients were hospitalized for a longer duration and resulted in the purchase of more medicine. Episiotomy increased expenditures for both uncomplicated NVD (by 37%) and eclamptic NVD (by 84%) compared to cases where no episiotomy was performed (data not shown). The medical reason for performing episiotomies is the prevention of perineal tearing but because of a high case load at DMCH physicians perform episiotomies to reduce the length of delivery time, effectively turning hospital expenditures into patient expenditures. *Eclampsia *increased the expenditures for NVD by 180% (data not shown). Eclampsia is not under the control of the health system or the patient, and procedures used for treating eclampsia are unavoidable.

C-sections caused higher patient expenditures compared to NVDs (median $119 and $63 respectively) because C-sections had a longer duration of hospitalization and required more medicine. Elective C-sections and eclamptic C-sections incurred similar expenses because elective C-sections were hospitalized for a longer duration even though there were no complications. Vaginal hysterectomies were 25% less expensive than abdominal hysterectomies because they required less invasive procedures, used local anaesthesia, and had a shorter duration of hospitalization.

### Sources of funds for patient expenditures

The respondents said that they were willing to pay for care. However, rural households reported more difficulty in paying for care than urban households. Difficulty was inferred from the number of households who borrowed to pay for care, and the ratio of the amount borrowed to the annual household income. The median patient expenditure was equivalent to 7% (range 0.04%–225%) of annual household income, and was higher for rural (median 10%; range 1%–225%) than urban (median 7%; range 0.04%–78%) respondents. Half (n = 40) of the households reported borrowing to pay for care. The patient who spent 225% of her annual household income was a rural patient who had a hysterectomy for prolapsed uterus. Surgical patients like her are usually hospitalized for a month as they require more tests than non-surgical patients. This patient's total expenditures were not higher than the others who also had a hysterectomy, however, her annual household income was much lower than that of the others.

More urban (n = 23) households borrowed than rural (n = 17) households but the amount borrowed was higher for rural households. The median amount borrowed per household was $38 and was equivalent to 8% (range 0.58%–208%) of the annual household income. On average, the rural households (median 14%; range 2%–208%) borrowed almost double the amount than the urban households (median 6%; range 0.58%–28%). Most often (n = 30) money was borrowed from friends and relatives without interest. When borrowing was from money-lenders (n = 10), households reported interest rates of 5%–30% per month. Three households put up security such as jewelry, land and household goods when borrowing money from moneylenders. The highest reported percentage of money borrowed to income for a rural household was 208% compared to 28% for an urban household. Finally, greater amounts of money were borrowed by C-section and hysterectomy patients than the other categories of patients. Table 3 illustrates the duration of hospitalization, total patient expenditures, annual household income, and amount borrowed by type of residence.

**Table 3:** Distribution of median duration of hospitalization, expenditures, income, and amount borrowed by residence

	**Urban**	**Rural**
**% of patients**	56% (n = 45)	44% (n = 36)
**Duration of hospitalization (day)**	7 (1–33)	9 (1–34)
**Total patient expenditures (US$)**	59.25 (2.15–350)	79.25 (2.40–250)
**Annual HH income (US$)**	900 (150–6000)	615 (2.50–6000)
**Borrowed amount (US$)**	37.50 (6.25–250)	52.50 (12.50–200)

Parenthesis shows range

HH: household

## Discussion and conclusions

The study findings indicate that all the surveyed patients incurred substantial out-of-pocket expenditures for a one time hospitalization. The median per patient expenditure was $65, and two-thirds of these expenditures were for commodities and services that were supposed to be provided free by the hospital but were not. Half the households borrowed to pay for care since they did not have the ability to pay (a finding similar to those found by Nahar et al.). A third of these households sold jewelry, land or household items to moneylenders. The rural households reported more difficulty in paying for care than the urban households. The rural patients had lower income but incurred higher expenditures and borrowed larger amounts than the urban patients.

The study data are a decade old but worth presenting because Bangladesh is only recently beginning health sector reform and fee for service. Also, the hospital practices with regard to providing maternity care remain the same, in the hospital studied and in other public hospitals in Bangladesh. Other limitations of the study are its small sample size and that all the interviewees came from only one hospital. The small sample size is the norm when doing in-depth interviews. When a paper is qualitative not quantitative no statistical tests are customarily done. The results are striking despite the limitations. The costs related to NVD and C-section were much higher in this study than that estimated by Nahar et al. (NVD: $62 and $32 respectively; C-section: $133 and $118 respectively). Possible reasons for the differences are: a) recall error higher in the Nahar et al. study as they interviewed post-partum mothers, whereas, this study interviewed patients during hospitalization; b) the Nahar et al. study included only uncomplicated cases, whereas, this study included both uncomplicated and complicated cases. The annual household income was lower in this study compared to the Nahar et al. study ($750 and $1476 respectively). This may be attributable to having rural patients in this study whose income is much lower than urban patients, whereas, Nahar et al. studied only urban patients.

Changing current practices regarding the length of hospitalization and medical procedures (e.g. episiotomy) will reduce patient expenditures. This can be achieved by having shorter duration of hospitalization for elective C-section and elective hysterectomy, and limiting the use of episiotomy. Limiting these practices may also lead to lower provider expenditures. Medicine constituted almost half of the total patient expenditures. However, lack of access to medicine resulted from an inefficient management system not from unavailability. Taking less time to process orders would make medicine available quicker to patients and so reduce their expenditures.

Hospital management needs to ensure that patients pay only the official rate of admission fees. Hospital management also needs to ensure that patients do not pay tips to salaried hospital staff for routine services. The hospital could arrange for liquid food and hot water for surgical patients in addition to the other services it already provides, i.e. provide a more comprehensive hotel service. Alternatively the hospital could subcontract out its hotel services to those employees who extract tips from patients to provide such services. This may act as an incentive for eliminating the unofficial fees from tips. Making some services such as eclampsia and hysterectomy available at secondary level facilities will benefit the rural patients by reducing the travel expenses. Government hospitals can generate revenue by introducing or increasing some fees for medicine and tests for the paying category of patients but clearly exempting the poorest. How would the system determine who was the poorest? One option would be to let all rural women use hospital for free and have all urban women pay some fee.

For service fees not to become a serious barrier to use current systems will have to eliminate or reduce practices that already result in high user expenditures from unofficial payments. Otherwise the added fees may lead to more borrowing from moneylenders, putting lands and goods at risk, and potential impoverishment of more households. A fee for service system needs to be based on information, and more than just setting a price. This can be facilitated by knowing what the patients were already spending in the current system and who were the most at risk of impoverishment. The challenge is to focus on realistic, short term changes that can reduce patient expenditures and inequities. Most of the recommendations of this study depend more on the will of the physicians and the hospital administrators than on the infusion of resources per se.

Free maternity services in Bangladesh impose large out-of-pocket expenditures on patients. Authorities could reduce the burden by reducing the duration of hospital stays, limiting use of medical procedures, eliminating tips, and moving routine services closer to potential users. Fee for service could reduce unofficial expenditures if the fee were lower than and replaced typical unofficial expenditures, otherwise adding service fees without reform of current hospital practices would lead to even more burdensome expenditures and inequities.

## Competing interests

The author(s) declare that they have no competing interests.
